# Very short answer questions vs. multiple choice questions in health professions education: a systematic review and meta-analysis

**DOI:** 10.1186/s12909-026-09359-5

**Published:** 2026-05-01

**Authors:** Ali Sher, Muhammad Moin Ud Din Arshad, Mesum Abbas, Abdul Hannan Asghar, Anees Hussain Khan, Junaid Ali, Muhammad Ibrahim Yaqoob, Rana Aftab Alam, Muhammad Bilal Akram, Muhammad Abdullah Masood, Amit Kumar Thakur

**Affiliations:** 1 Quaid-e-Azam Medical College, Bahawalpur, Pakistan; 2 Shahida Islam Medical College, Lodhran, Pakistan; 3 Al-Nafees Medical College, Islamabad, Pakistan; 4 Sukrajaj Hospital of Tropical and Infectious Disease, Teku, Kathmandu Nepal

**Keywords:** Very Short Answer Questions, Multiple Choice Questions, Cueing Effect, Assessment Reliability, Discrimination Index, Medical Education, Meta-analysis, Psychometrics.

## Abstract

**Background:**

Multiple Choice Questions (MCQs) remain the most widely used written assessment format in health professions education due to their efficiency and reliability; however, their susceptibility to cueing raises concerns regarding their authenticity in assessing true knowledge. Very Short Answer Questions (VSAQs) have emerged as a promising alternative that reduces cueing effects and requires learners to generate responses with greater cognitive engagement, while retaining the logistical advantages of computer-marked assessments. This systematic review and meta-analysis evaluates the comparative performance, discrimination, reliability, and practice effects of VSAQs versus MCQs.

**Methods:**

Following PRISMA guidelines (31), a systematic search identified studies comparing VSAQs and MCQs in undergraduate and postgraduate health professions education. Data extraction and risk of bias assessment were conducted independently by two reviewers, with disagreements resolved through consensus. Mean scores, standard deviations, discrimination indices, and reliability coefficients were extracted. Standardized Mean Differences (SMD) were pooled using a random-effects model. Psychometric indices were transformed using Fisher’s z-scores. Heterogeneity was quantified using the I² statistic, and sensitivity and subgroup analyses were performed to explore the impact of outliers.

**Results:**

Six cohorts (*n* = 1,191) derived from three included studies contributed data for score comparisons. The initial pooled analysis showed no significant difference between VSAQs and MCQs (SMD = − 0.52; 95% CI − 1.34 to 0.30), with extreme heterogeneity (I² = 98%). Sensitivity analysis identified Dhok et al. (2023) as an outlier. Excluding this dataset yielded a significant effect favoring MCQs (SMD = − 0.86; 95% CI − 1.01 to − 0.70; *p* < 0.00001) with markedly reduced heterogeneity (I² = 4%). Meta-analysis of psychometric properties demonstrated strong discrimination (pooled Fisher’s z = 1.45) and acceptable reliability (pooled Fisher’s z = 0.43) for VSAQs. Evidence on practice effects was mixed, with no consistent advantage for either format.

**Conclusion:**

VSAQs exhibit strong psychometric integrity and are designed to reduce the cueing effect observed in MCQs. Although associated with lower student scores, VSAQs likely provide a more accurate reflection of independent knowledge and clinical reasoning ability. Their integration into medical assessment systems may enhance the authenticity and validity of written examinations.

## Highlights

Students generally scored lower on VSAQs than MCQs, consistent with reduced cueing effects and greater cognitive engagement required for self-generated responses.

VSAQs demonstrated high discrimination and acceptable reliability, comparable to or exceeding MCQs, indicating strong psychometric performance.

Sensitivity analysis revealed that removing a single statistical outlier (Dhok et al., 2023) reduced heterogeneity from 98% to 4%, yielding a robust pooled effect favoring MCQs.

VSAQs reduce reliance on recognition-based answering, offering more reliable assessment of independent knowledge relevant to clinical reasoning.

Practice effects for VSAQs remain inconclusive, with mixed findings across studies, highlighting the need for further research on retrieval-based learning benefits.

## Introduction

Assessment is a central component of health professions education, shaping both learning and professional competence. The quality of assessment design directly influences the validity of score interpretation, learner progression, and high-stakes decisions within training programs [1]. Multiple Choice Questions (MCQs) remain the predominant written assessment format due to their efficiency, ease of scoring, and perceived reliability. MCQs are widely adopted for both formative and summative evaluation in medical education globally [1, 2]. Despite their advantages, MCQs are susceptible to cueing effects, where recognition rather than recall may drive performance, potentially inflating student scores without truly reflecting independent knowledge [3, 4]. Alternative formats, such as Very Short Answer Questions (VSAQs), have been proposed to mitigate cueing by requiring concise, self-generated responses [5–7].There is a growing shift within medical education toward reassessing traditional MCQ-based formats. Several institutions and assessment bodies have begun incorporating constructed-response formats to better align examinations with higher-order competencies and clinical reasoning. For example, the National Board of Medical Examiners has emphasized the importance of testing application and reasoning beyond factual recall [8]. VSAQs are particularly appealing because they retain many logistical benefits of MCQs, including computer-markable formats and rapid scoring, while reducing cueing effects and demanding greater cognitive engagement through self-generated responses [9–11]. Previous studies suggest that VSAQs may enhance assessment authenticity, improve discrimination between high- and low-performing students, and more accurately evaluate clinical reasoning, [12–14]. The theoretical rationale for constructed-response assessments is supported by cognitive psychology principles, including retrieval practice and test-enhanced learning, which suggest that generating answers with minimal cueing may strengthen retention and deepen understanding compared with recognition-dominant formats [15–17]. Despite these advantages, evidence regarding the psychometric performance and comparative effectiveness of VSAQs versus MCQs remains limited and fragmented. Systematic investigations have reported mixed findings regarding reliability, discrimination, and practice effects, [9, 18, 19]. Furthermore, the acceptability of VSAQs among students and educators, as well as their integration into existing curricula, is an ongoing concern, [20–22]. The need for a comprehensive synthesis of the literature is critical to guide educators on the optimal implementation of assessment formats that balance feasibility with validity.

An important consideration is the applicability of these findings to examinations that combine both MCQs and VSAQs, which reflects common practice in many health professions programs. While MCQs allow efficient assessment of broad knowledge and are associated with higher scores, VSAQs demonstrate strong discrimination and acceptable reliability, supporting their role in assessing knowledge with reduced cueing and greater response precision [28–30]. Integrating both formats may therefore provide a balanced assessment strategy that enhances validity while maintaining feasibility.

This systematic review and meta-analysis therefore aims to evaluate the comparative performance, psychometric properties, and practical implications of VSAQs versus MCQs in health professions education. Specifically, we examine differences in student scores, discrimination indices, reliability coefficients, and the impact of practice effects, thereby providing an evidence-based framework for assessment design in medical and allied health education, [23–30].

## Methodology

### Study design and data synthesis

This systematic review and meta-analysis was conducted to evaluate the comparative performance and psychometric properties of Very Short Answer Questions (VSAQs) versus Multiple Choice Questions (MCQs) in health professions education. The study adhered to the Preferred Reporting Items for Systematic Reviews and Meta-Analyses (PRISMA) guidelines [30]. Inclusion and exclusion criteria were established prior to study selection.

The risk of bias for included studies was assessed using the Cochrane Risk of Bias tool as implemented in Review Manager (RevMan). Bias was evaluated across the following domains: random sequence generation, allocation concealment, blinding of participants and personnel, blinding of outcome assessment, incomplete outcome data, and selective reporting. For non-randomized studies included in the analysis, selection bias domains were adjudicated based on study design constraints, noting where randomization was not applicable. To minimize bias, missing data were first sought by contacting study authors. When data could not be obtained, such studies were excluded from pooled statistical analysis but were narratively described where applicable.

The risk of bias assessment, conducted using the Cochrane Risk of Bias tool, revealed a variable risk profile across the included studies. Risk of bias assessment was conducted independently by two reviewers, with disagreements resolved by consensus.

- Performance Bias: All primary studies were assessed as having a High Risk of performance bias. Due to the nature of the educational intervention, it was impossible to blind participants to the question format they were attempting.
- Detection Bias: The risk of detection bias was generally Low for the majority of studies (Sam et al., 2019; van Wijk et al., [16]; Potter & McLachlan, 2025; van Wijk et al., 2024), which utilized machine marking or blinded panel reviews. However, Dhok et al. (2023) was assessed as High Risk for this domain because VSAQs were marked manually by the investigators, introducing potential subjectivity.
- Selection Bias: Randomized studies demonstrated a Low Risk for sequence generation and allocation concealment. Non-randomized cohort studies were assessed based on their specific design constraints, with some inherent selection bias noted in voluntary participation models.

**Fig. 1:**
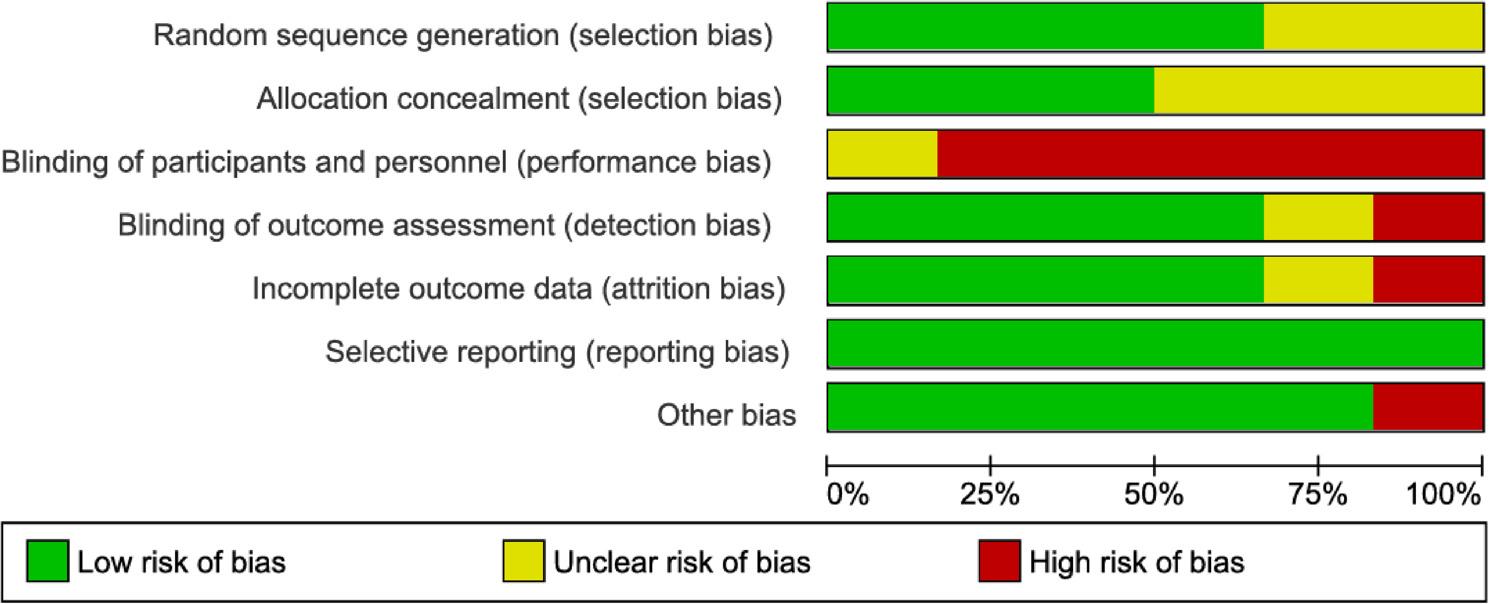
Risk of Bias Graph: Review authors' judgments about each risk of bias item presented as percentages across all included studies. Performance bias was assessed as high risk across all studies due to the impossibility of blinding participants to the question format

**Fig. 2:**
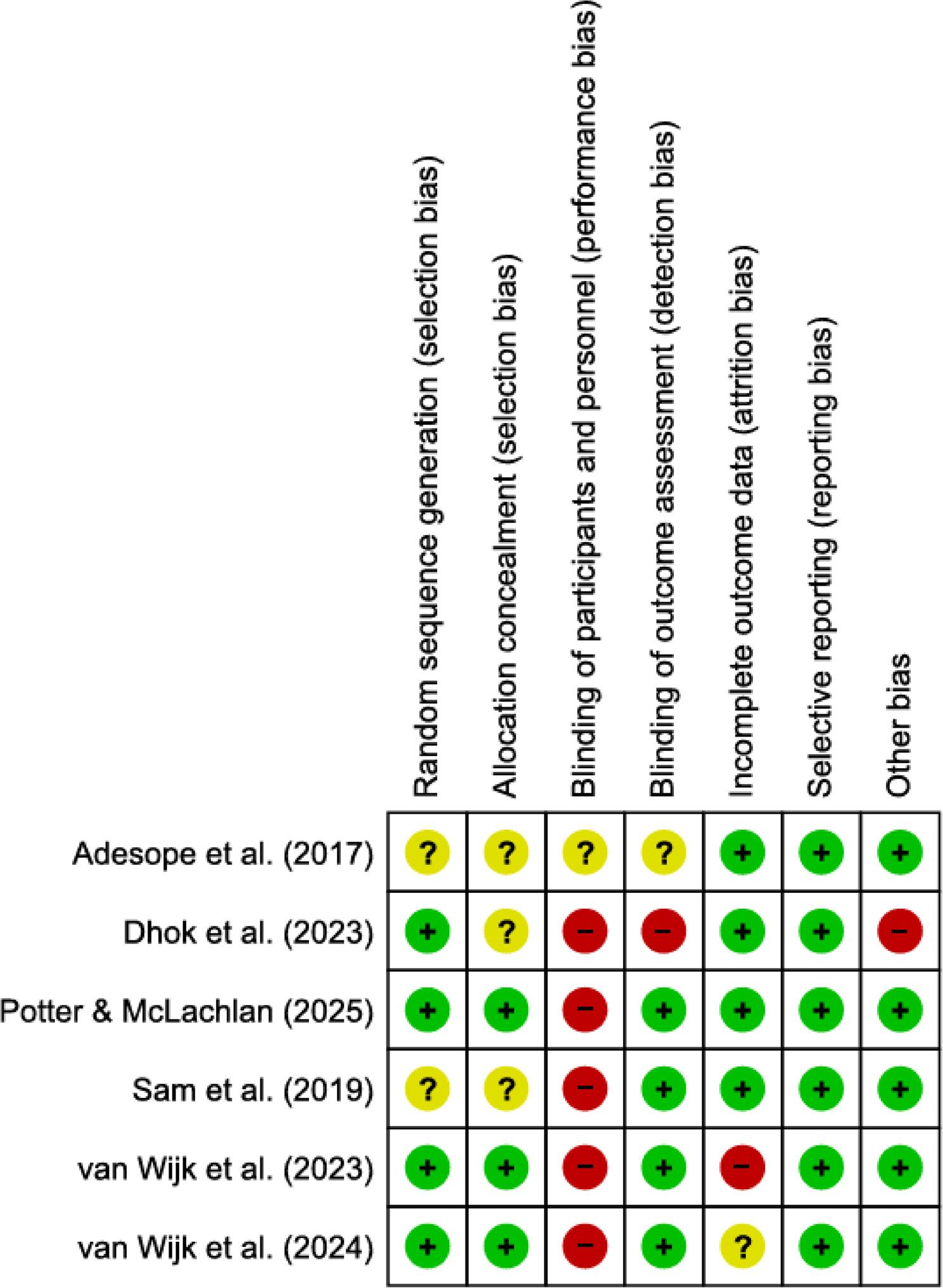
Risk of Bias Summary: A detailed overview of the review authors' judgments about each risk of bias item for each included study

The risk of bias graph and summary are given in Figs. 1 and 2, respectively.

### Outcomes and effect measures

The primary outcome was student performance (scores). Secondary outcomes included psychometric indices: discrimination and reliability.

- Assessment Scores: Continuous data (mean scores and standard deviations) were extracted. The effect size was calculated as the Standardized Mean Difference (SMD) using a Random-Effects model due to anticipated variance in assessment difficulty and contexts across institutions.
- Psychometric Properties: Reliability and discrimination coefficients reported in included studies were converted to Fisher’s z-scores to allow for stabilization of variance and valid pooling.
- Practice effects: Practice effects were explored as an additional outcome where data were available. Due to limited and heterogeneous data, this analysis was considered exploratory.

### Statistical analysis

All analyses were performed using Review Manager (RevMan) 5.4. A Random-Effects model was applied for all meta-analyses. Heterogeneity was assessed using the Chi^2^ test and the I^2^ statistic; values of 25%, 50%, and 75% were interpreted as low, moderate, and high heterogeneity, respectively.

To address significant heterogeneity observed in the primary outcome, a pre-specified sensitivity analysis was conducted. Studies identified as statistical outliers (visualized via forest plots and confirmed by substantial deviation from the pooled mean) were removed to calculate a robust estimate of the true effect size. Subgroup analyses were performed to distinguish between “Main Studies” and “Outlier” datasets.

### Eligibility criteria

#### Inclusion criteria 

Studies were included in this systematic review if they met the following criteria:

- Population: Undergraduate or postgraduate students enrolled in health professions education programs (e.g., Medicine, Nursing, Dentistry, Allied Health).
- Intervention & Comparator: Studies directly comparing student performance or psychometric properties between Very Short Answer Questions (VSAQs) (defined as open-ended questions requiring a self-generated response of 1–4 words) and Multiple Choice Questions (MCQs) (Single Best Answer format).
- Outcomes: Studies reporting extractable quantitative data, specifically:
  - Student Performance: Mean assessment scores and standard deviations (SD).
  - Psychometrics: Reliability coefficients (e.g., Cronbach’s alpha) or discrimination indices (e.g., point-biserial correlation).
- Study Design: Randomized controlled trials (RCTs), crossover trials, or comparative cohort studies.

#### Exclusion criteria

Studies were excluded if they:

- Intervention Mismatch: Compared MCQs to long-form essay questions, oral examinations, or fill-in-the-blank questions that did not meet the definition of VSAQs (computer-markable, limited word count).
- Insufficient Data: Reported only qualitative feedback (e.g., student preference surveys) without objective performance metrics or psychometric data.
- Publication Type: Were conference abstracts, reviews, editorials, or studies where full-text data was unavailable.
- Data Structure: Studies where the VSAQ and MCQ components assessed completely different content domains, preventing valid comparison.

**Fig. 3:**
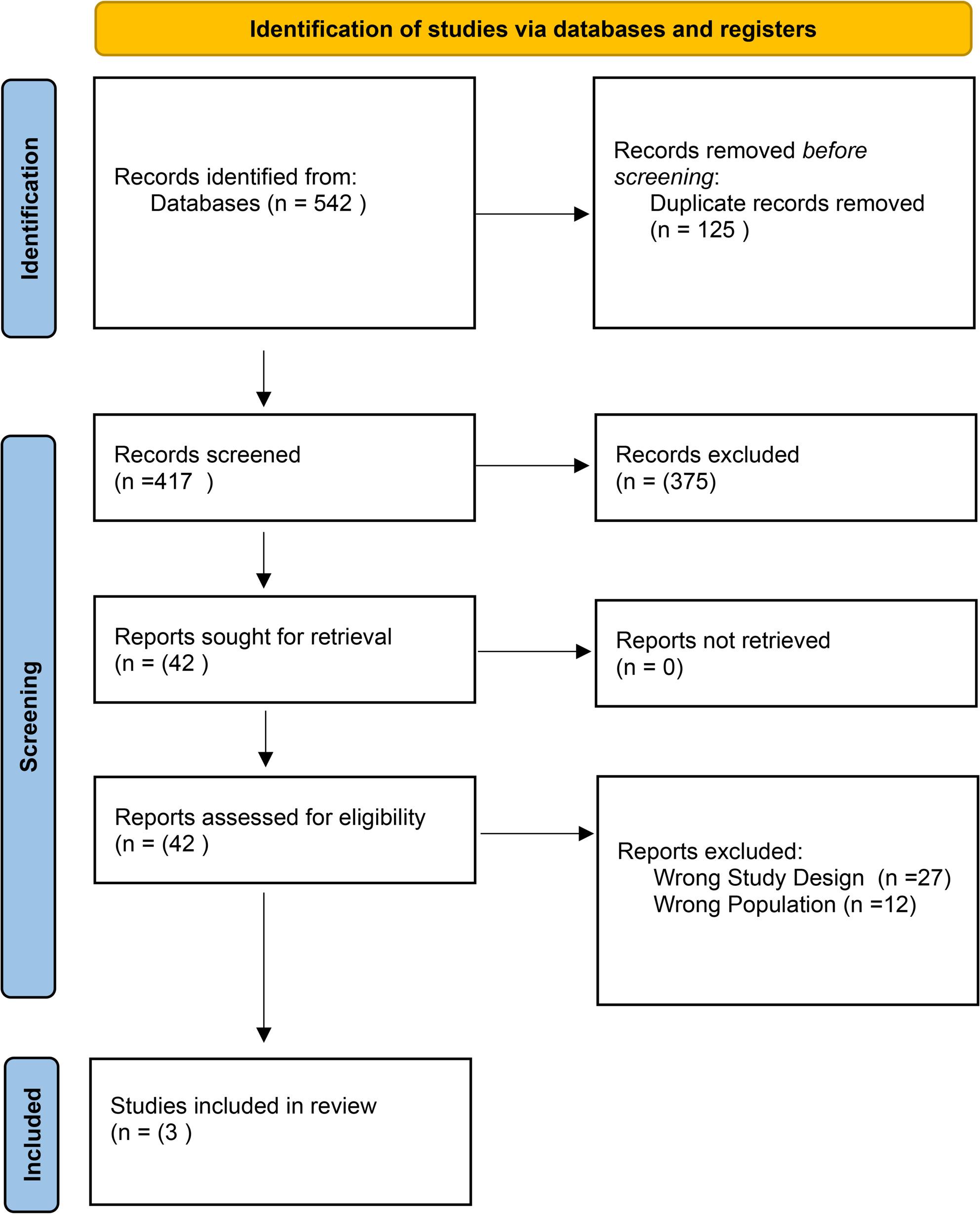
PRISMA 2020 Flow Diagram [31] depicting the systematic search strategy, screening process, eligibility assessment, and final inclusion of studies for the review

A PRISMA Flowchart is given in Fig. 3.

## Results

1. Comparison of assessment scores (Performance)

A total of six cohorts from three studies (Dhok et al., 2023; Potter & McLachlan, 2025 [Year A, B, C]; van Wijk et al., [16] [DA, RM]) were included in the quantitative synthesis for assessment scores (Total No = 1,191).

**Fig. 4:**
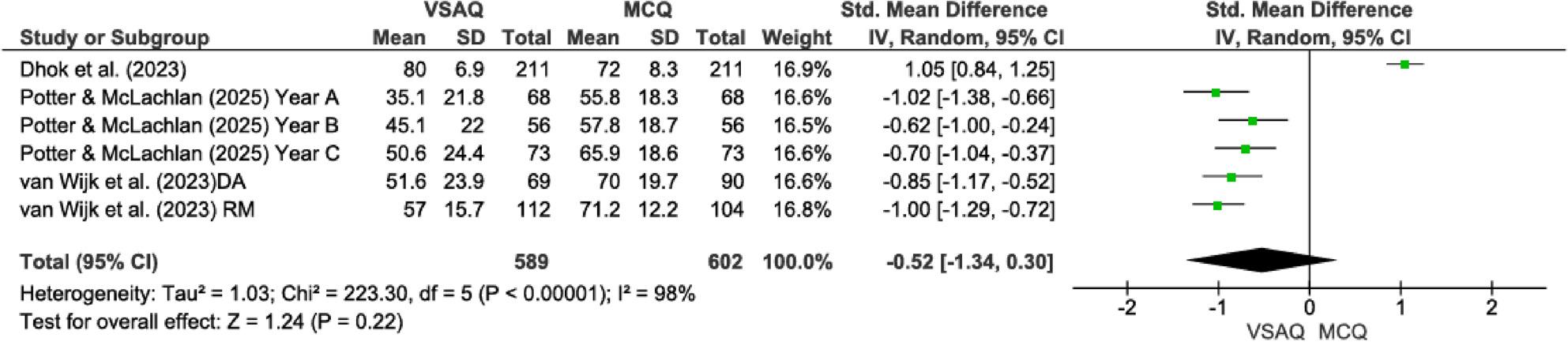
Forest plot comparing student assessment scores between VSAQs and MCQs (Initial Pooled Analysis). The analysis includes all six cohorts and demonstrates no statistically significant difference (SMD = –0.52) but exhibits extreme heterogeneity

**Fig. 5:**
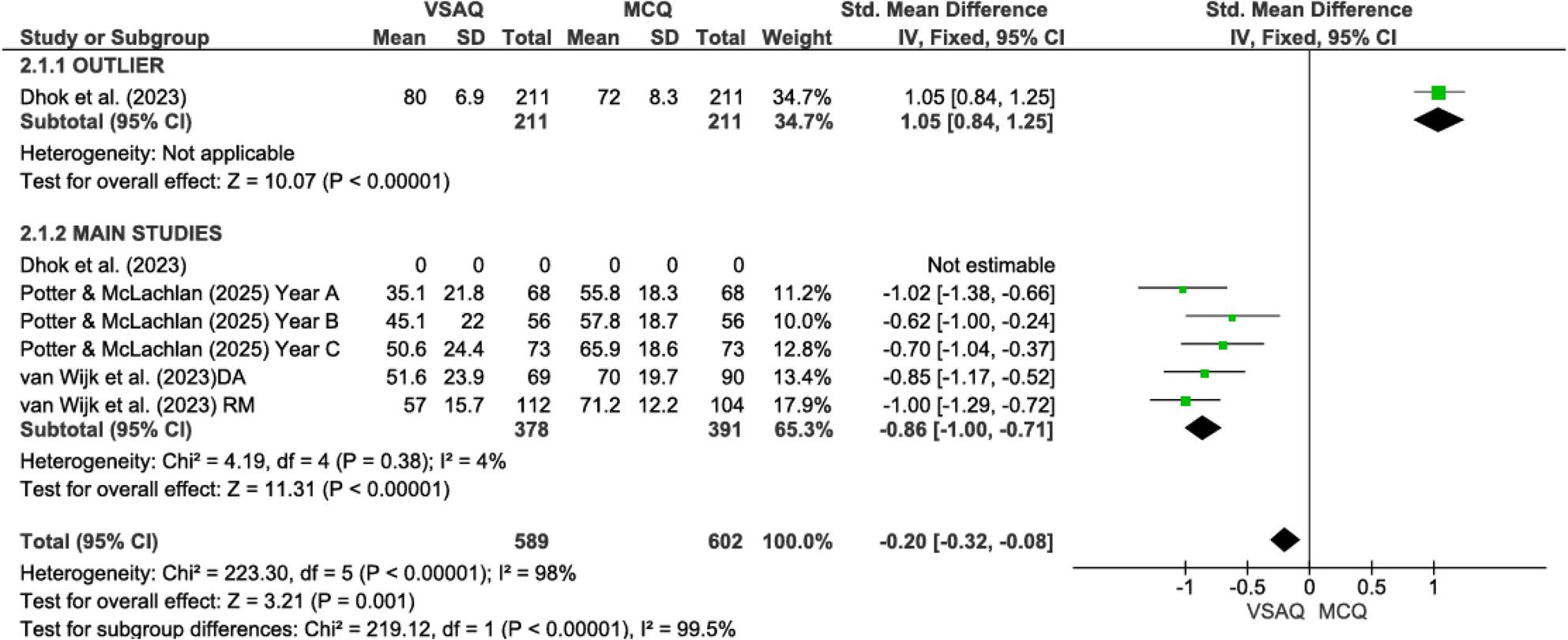
Forest plot illustrating the sensitivity and subgroup analysis. The plot visually identifies Dhok et al. (2023) as a significant outlier favoring VSAQs (SMD > 1.0), while all other cohorts consistently favored MCQs

**Fig. 6:**
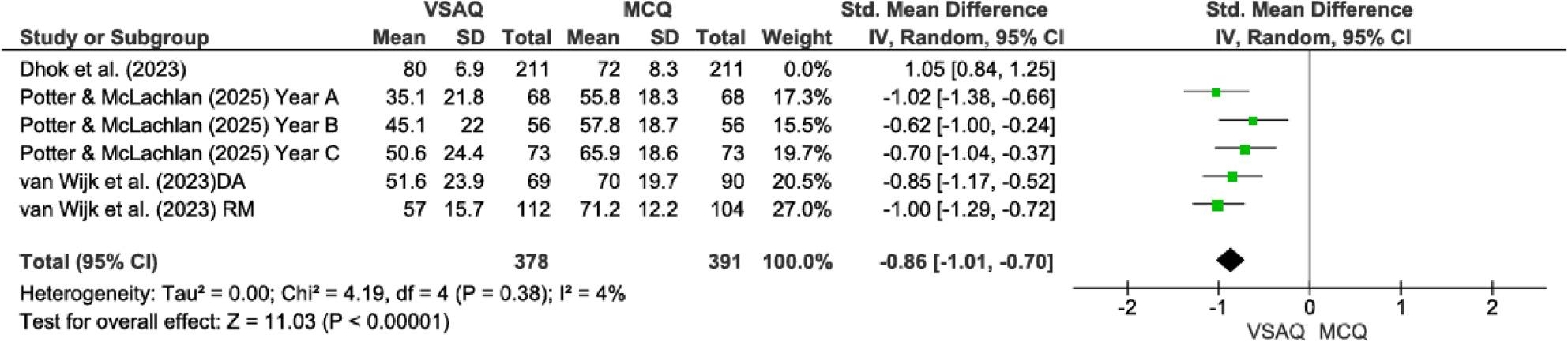
Forest plot of student assessment scores excluding the statistical outlier (Dhok et al., 2023). The robust pooled estimate shows a statistically significant difference favoring MCQs (SMD = –0.86) with markedly reduced heterogeneity

- Overall Analysis: The initial pooled analysis revealed no statistically significant difference in students’ performance between VSAQ and MCQ scores (SMD = -0.52; 95% CI [-1.34, 0.30]; *p* = 0.22). However, this analysis suffered from extreme heterogeneity (I^2^ = 98%, *p* < 0.00001), indicating that the pooled result was influenced by inconsistent study outcomes. (Fig. 4)
- Sensitivity and Subgroup Analysis: Visual inspection of the forest plot identified Dhok et al. (2023) as a significant outlier. While Dhok et al. reported higher scores in the VSAQ format (SMD = 1.05 [0.84, 1.25]), all other cohorts (Potter & McLachlan; van Wijk et al.) consistently showed higher performance in the MCQ format. (Fig. 5)
- Main Effects (Outlier Removed): After excluding the outlier (Dhok et al.), the sensitivity analysis (*n* = 5 cohorts) demonstrated a statistically significant difference favoring MCQs. The pooled Standardized Mean Difference was **−** 0.86 (95% CI [-1.01, -0.70]; *p* < 0.00001), indicating that students scored significantly lower on VSAQs compared to MCQs. The removal of the outlier successfully reduced heterogeneity from 98% to 4% (*p* = 0.38), confirming the robustness of this finding across the main studies. (Fig. 6)

2. Psychometric properties

To ensure the validity of the score comparisons, the psychometric quality of the assessments was evaluated via meta-analysis of discrimination and reliability indices.

**Fig. 7:**
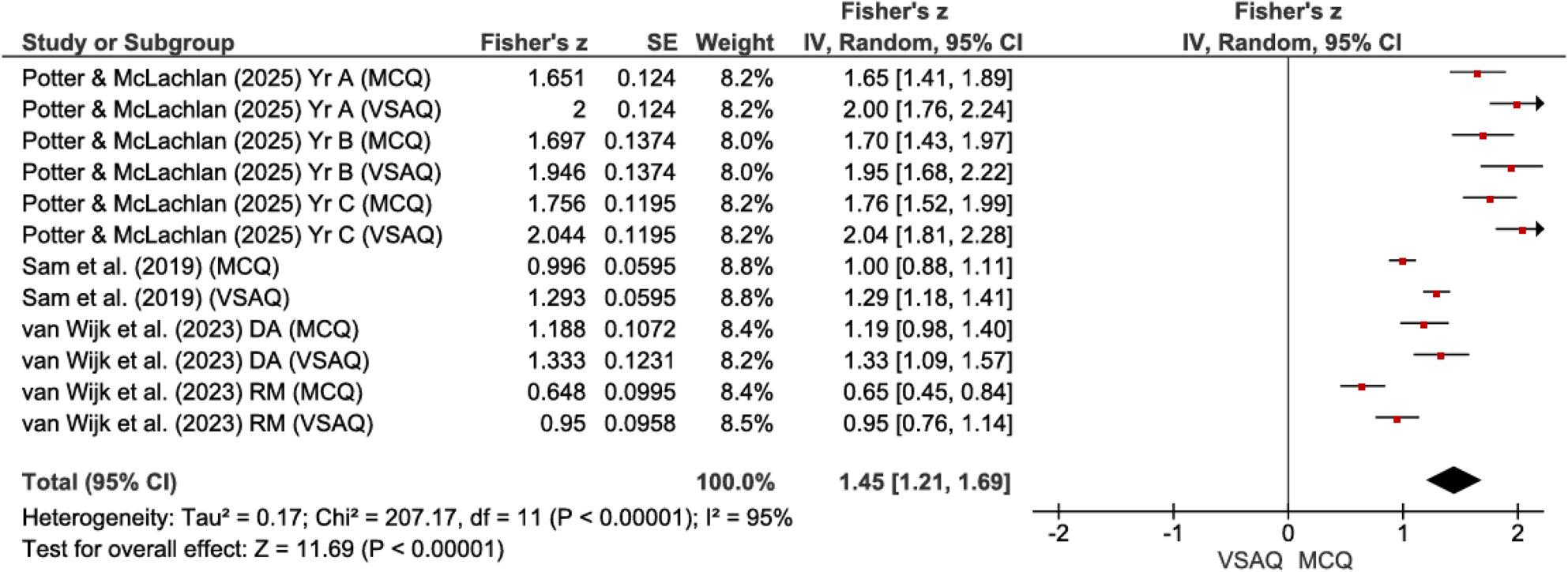
Forest plot of the discrimination indices (Fisher’s z-transformed) comparing VSAQs and MCQs. The pooled estimate of 1.45 suggests strong discriminatory power for VSAQs, though high heterogeneity indicates variance across study contexts

**Fig. 8:**
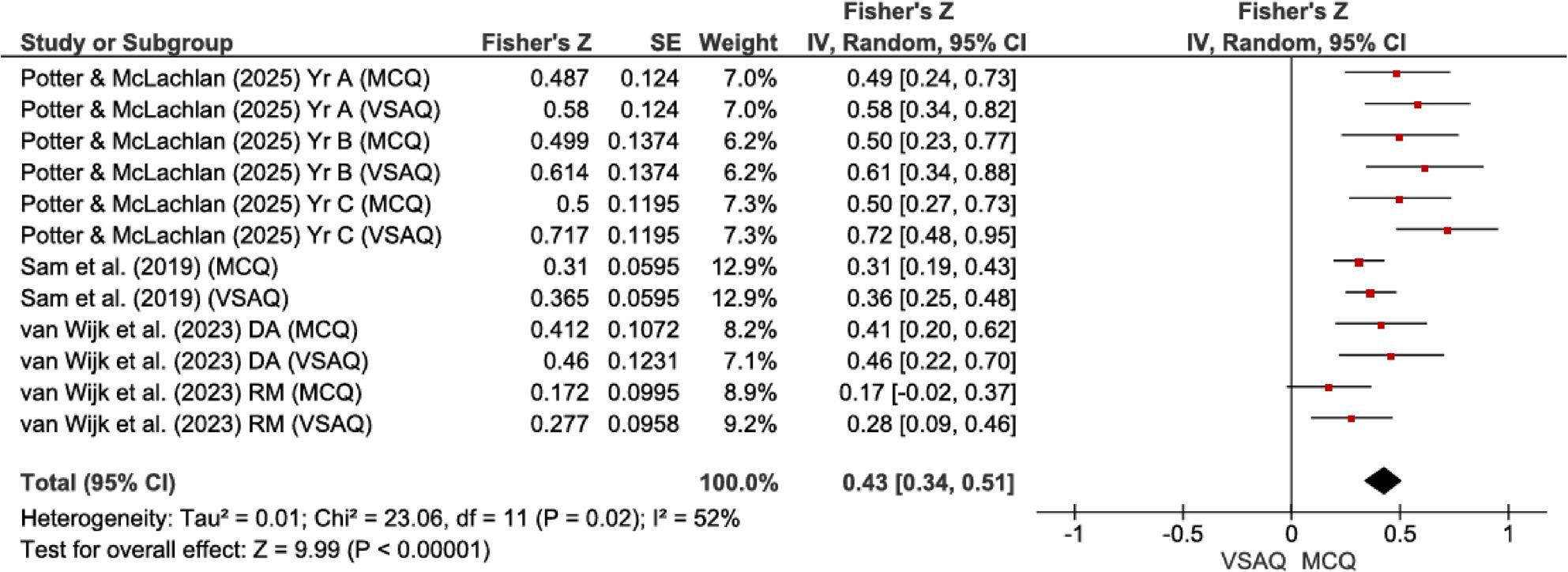
Forest plot of reliability indices (Fisher’s z-transformed) for VSAQs. The analysis yields a significant overall effect estimate of 0.43 with moderate heterogeneity, indicating acceptable internal consistency

- Discrimination: The meta-analysis of discrimination indices (Fisher’s z-transformed) across 12 data points yielded a pooled estimate of 1.45 (95% CI [1.21, 1.69]). This result was statistically significant (Z = 11.69, *p* < 0.00001), suggesting that the assessments included in the review generally demonstrated strong discriminatory power, though high heterogeneity was observed (I^2^ = 95%). (Fig. 7)
- Reliability: The pooled analysis of reliability indices (Fisher’s z-transformed) yielded a significant overall effect estimate of 0.43 (95% CI [0.34, 0.51]; *p* < 0.00001). Heterogeneity for reliability was moderate (I^2^ = 52%), indicating reasonable consistency in the internal consistency of the assessments used across the included studies. (Fig. 8)

### Practice effects

**Fig. 9:**
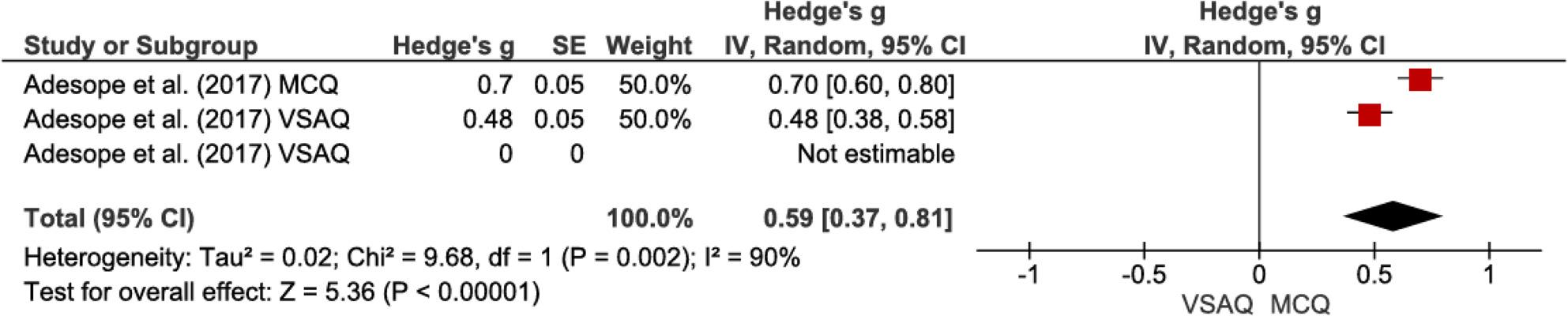
Forest plot comparing the practice effects (testing effect) of VSAQs versus MCQs. The results are mixed, showing no consistent statistically significant advantage for either format regarding retrieval-based learning benefits in recent data

3. Practice effects

- An analysis of practice effects (Adesope et al., 2017; van Wijk et al., 2024) showed mixed results. While earlier data (Adesope et al.) suggested a positive effect size favoring VSAQ practice (Hedges’ g = 0.59), more recent specific practice data (van Wijk et al., 2024) showed a non-significant trend (Hedges’ g = -0.26; 95% CI [-0.56, 0.03]; *p* = 0.08), limiting definitive conclusions regarding the superiority of one format for retrieval practice based on this dataset alone. (Fig. 9)

### Assessment of publication bias and heterogeneity

Visual inspection of funnel plots was conducted to assess the presence of publication bias and to identify sources of heterogeneity across the primary and secondary outcomes.

1. Student performance (Assessment Scores)

**Fig. 10:**
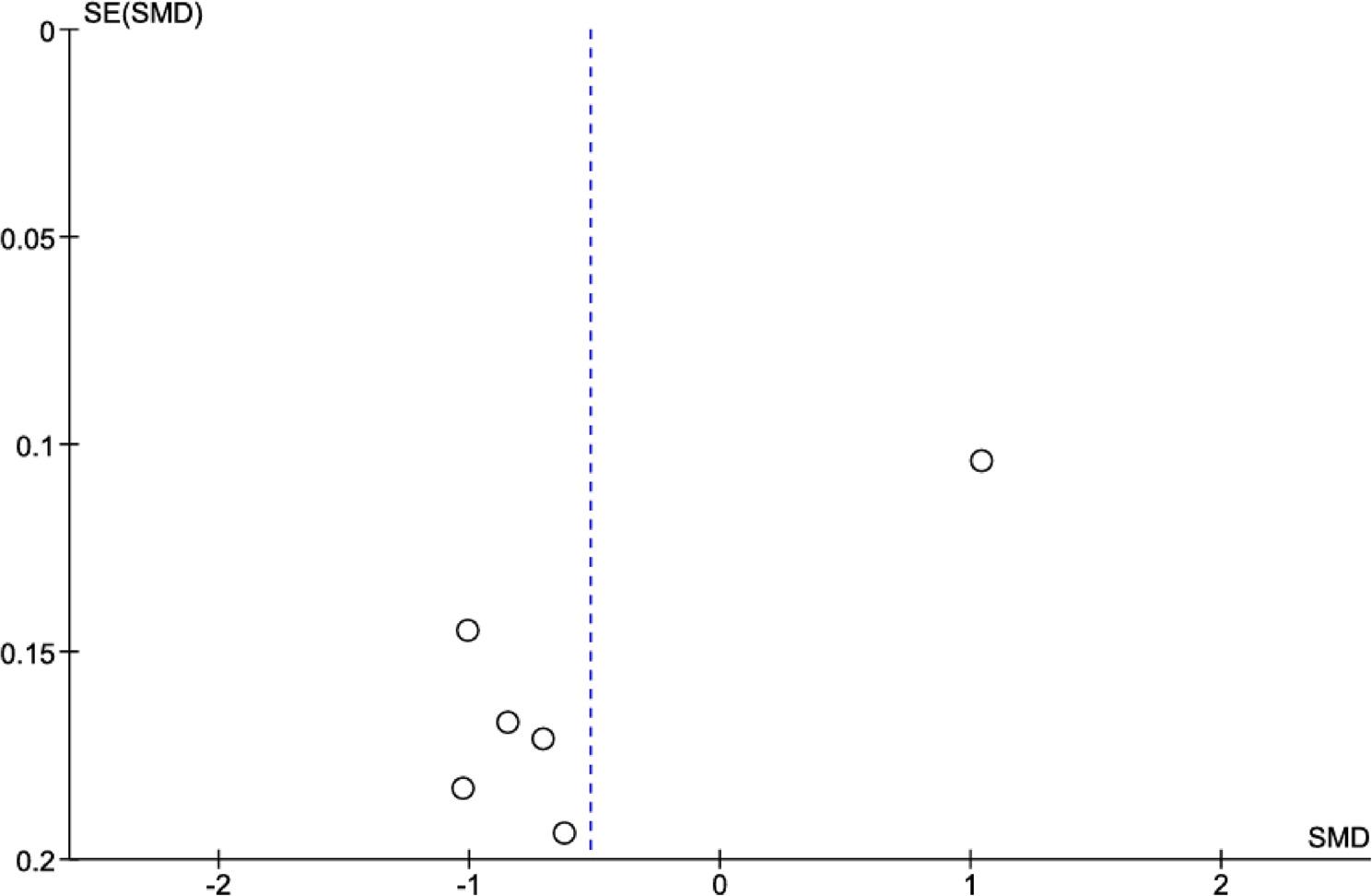
Funnel plot for the primary outcome of student assessment scores. The plot displays marked asymmetry with a distinct clustering of studies on the left (favoring MCQs) and a solitary statistical outlier (Dhok et al., 2023) on the far right, confirming the source of heterogeneity

The funnel plot for the primary outcome of student scores displayed marked asymmetry 1. A distinct clustering of studies was observed on the left side of the plot (negative Standardized Mean Difference), indicating that the majority of cohorts scored lower on VSAQs compared to MCQs. However, a solitary statistical outlier was identified on the far right (positive SMD > 1.0). This visual outlier corresponds to the study by Dhok et al. (2023), confirming it as the primary source of the extreme heterogeneity (I^2^ = 98%) observed in the initial pooled analysis. The lack of smaller, negative studies in the lower-left quadrant may also suggest a potential small-study effect. (Fig. 10)

**Fig. 11:**
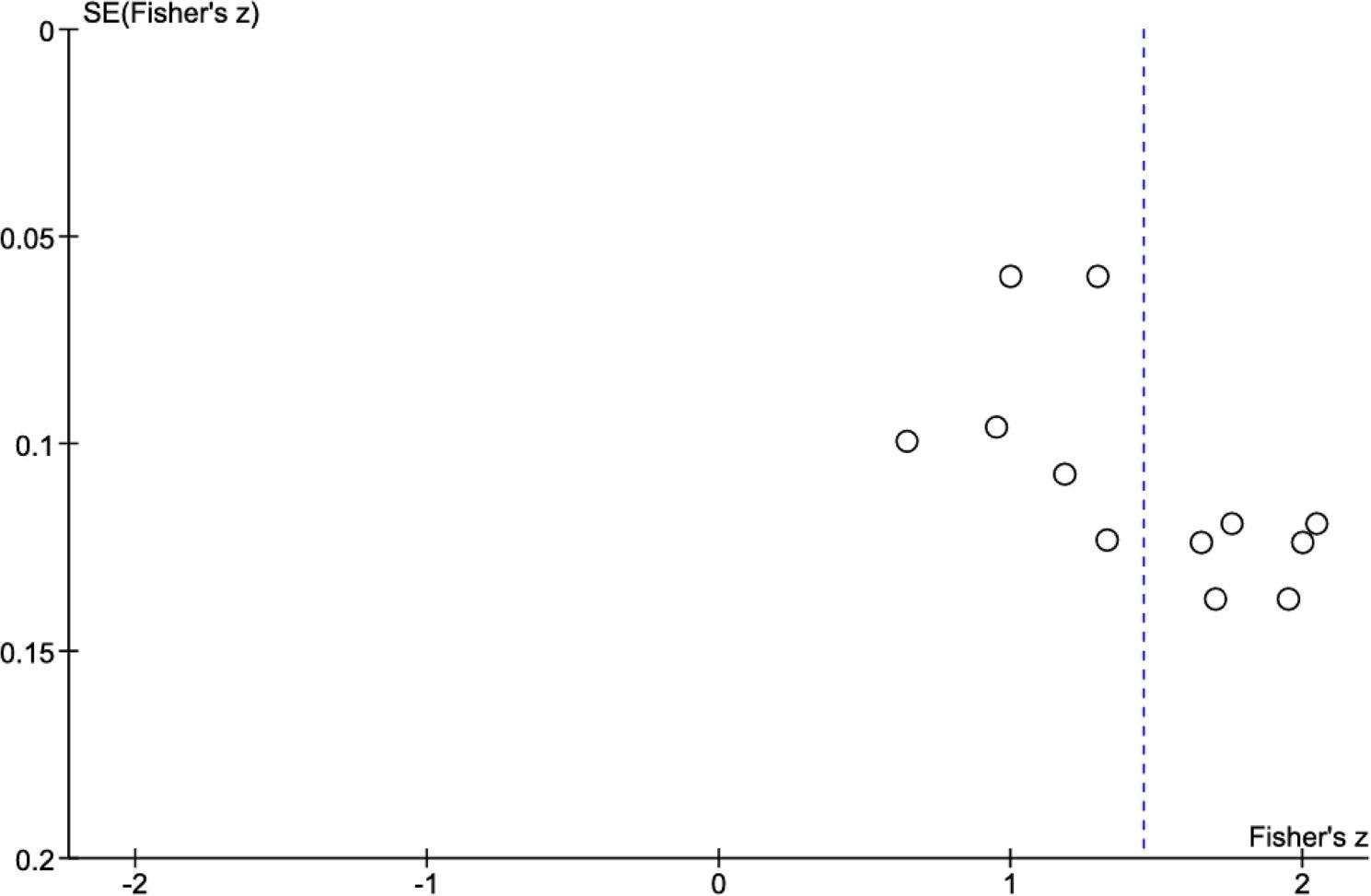
Funnel plot for discrimination indices. The wide scatter of data points reflects the high heterogeneity observed in the main analysis, suggesting that discriminatory power varies significantly by question design

**Fig. 12:**
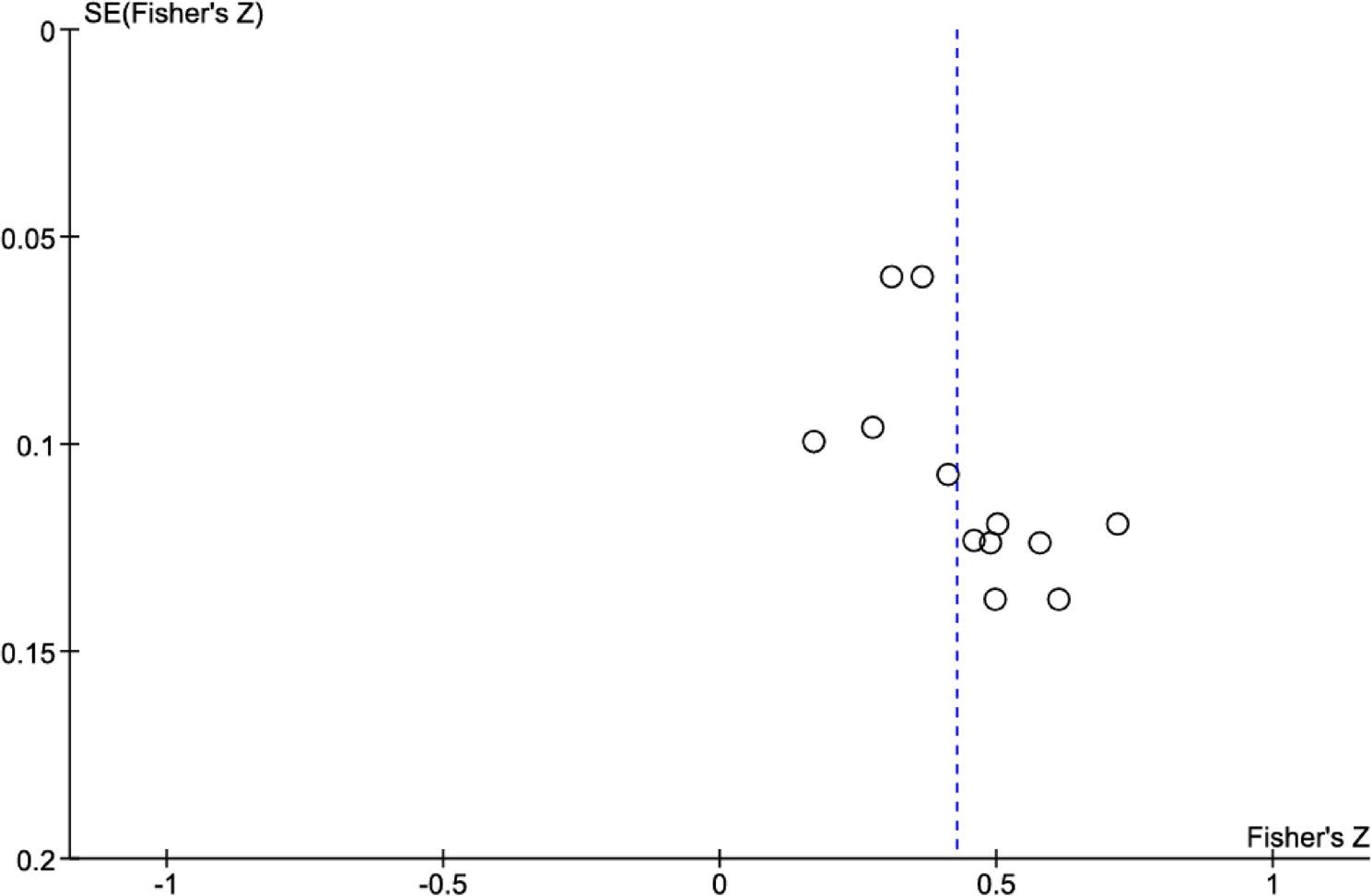
Funnel plot for reliability indices. The data points show a relatively tighter clustering around the pooled estimate compared to discrimination indices, indicating greater consistency in the reliability of VSAQs across studies

2. Psychometric properties

- Discrimination: The funnel plot for discrimination indices revealed a wide scatter of effect sizes (Fisher’s z) across the vertical axis. The data points did not converge into a typical funnel shape, reflecting the high heterogeneity (I^2^ = 95%) reported in the main analysis. This distribution suggests that the discriminatory power of VSAQs varies significantly depending on the specific context and question design of individual studies. (Fig. 11)
- Reliability: In contrast, the funnel plot for reliability indices showed a relatively tighter clustering around the pooled estimate (Fisher’s z ≈ 0.43). While some asymmetry persists, the distribution indicates more consistency in the internal consistency of VSAQs compared to their discrimination indices. (Fig. 12)

3. Practice effects

**Fig. 13:**
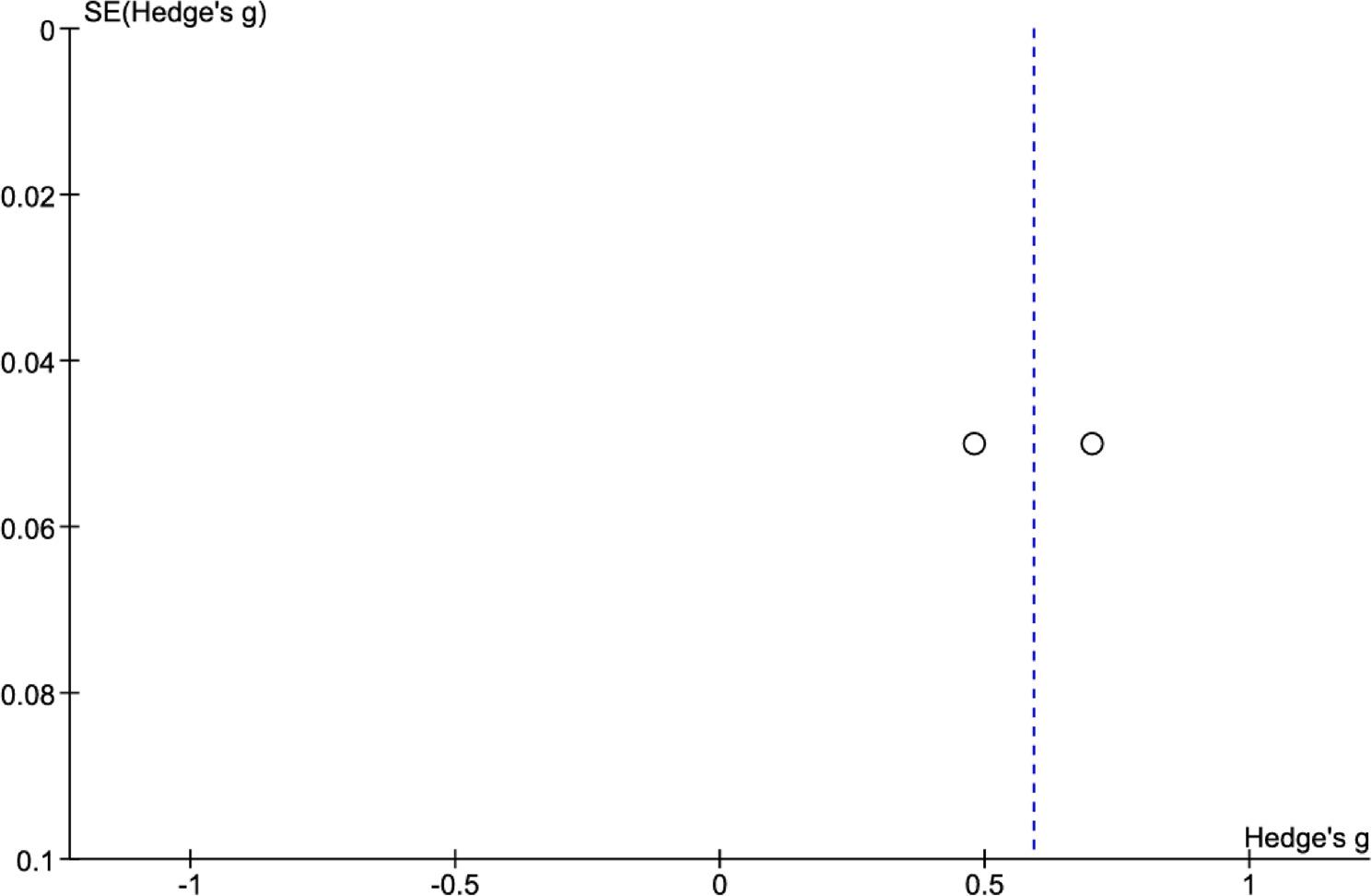
Funnel plot for practice effects. The plot is sparse due to the limited number of eligible studies included in the sub-analysis, preventing definitive conclusions regarding publication bias for the "testing effect" outcome

The funnel plot for practice effects was sparse due to the limited number of eligible studies included in this sub-analysis. Consequently, no definitive conclusions regarding publication bias could be drawn for the “testing effect” outcome, highlighting the need for further experimental research in this domain. (Fig. 13)

## Discussion

Our meta-analysis demonstrates that students generally score lower on VSAQs than on MCQs, consistent with reduced cueing effects and the greater cognitive demand required for self-generated responses [1, 5, 9, 11]. Importantly, both MCQs and VSAQs require retrieval from memory; the principal distinction is that VSAQs reduce response cueing and place greater demands on answer generation. This difference persisted even after sensitivity analyses, indicating a robust effect across institutions and cohorts. Lower scores on VSAQs likely reflect more authentic assessment of independent knowledge and clinical reasoning, rather than deficiency in learning [4, 6, 7]. These findings align with prior reports suggesting that MCQs may overestimate student mastery due to recognition-driven answering [3, 11, 13].

Psychometric evaluation of VSAQs indicated strong discrimination and acceptable reliability, comparable to or exceeding that of MCQs [8, 10, 14]. The pooled Fisher’s z-values confirmed that VSAQs effectively differentiate between high- and low-performing students, consistent with literature suggesting that constructed-response formats may serve as more sensitive indicators of learner competence when cueing is minimized [13, 16, 17]. The integration of VSAQs may therefore improve assessment validity without compromising scoring efficiency [18, 20].

Practice effects, however, remain inconclusive. Some studies suggest that repeated exposure to VSAQs may enhance retention through retrieval practice, consistent with cognitive theories of test-enhanced learning [15, 17, 21]. Retrieval-based assessment has been shown to enhance long-term retention through repeated retrieval efforts. Because VSAQs require learners to generate responses with fewer external cues, they may promote deeper encoding and more effortful retrieval than recognition-dominant formats [18, 20]. Others report minimal advantage over MCQs, highlighting variability in study design, sample size, and prior knowledge of students [19, 22, 23]. These mixed results underscore the need for careful consideration when incorporating VSAQs into longitudinal assessment strategies.

The adoption of VSAQs also addresses educational equity concerns. Open-ended, recall-based formats reduce the advantage of testwise students who excel primarily through recognition and strategic guessing in MCQs [1, 3, 7]. Moreover, their acceptability and feasibility have improved with advancements in automated marking systems, allowing for scalable implementation even in large cohorts [9, 10, 24, 25]. Nonetheless, faculty training and standard-setting remain essential to maintain scoring consistency and fairness [26, 27].

From a broader perspective, assessment design must align with learning objectives and intended competencies. Evidence from this review supports the integration of VSAQs for higher-order cognitive assessment while retaining MCQs for foundational knowledge testing, achieving a balanced evaluation strategy, [28–30]. In this context, VSAQs offer a practical and psychometrically sound complement to traditional MCQs, enhancing the authenticity of written examinations and promoting meaningful learning. At an institutional level, this approach could enhance assessment validity while maintaining feasibility in large cohorts. Additionally, these findings highlight the need for faculty development in writing high-quality VSAQs and investment in automated marking systems to ensure feasibility and consistency.

A summary of findings table is given in Table 1.

**Table 1:** Summary of Meta-analysis Findings Comparing Very Short Answer Questions (VSAQs) and Multiple-Choice Questions (MCQs) in Health Professions Education

Outcome Category	Key Findings & Statistical Results	Interpretation & Certainty
Student Performance (Assessment Scores)	Initial analysis: SMD = -0.52; I² = 98% (no significant difference; extreme heterogeneity). Sensitivity analysis (excluding outlier Dhok et al.): SMD = -0.86; 95% CI -1.01 to -0.70; *p* < 0.00001 (favors MCQs). Heterogeneity after exclusion: I² = 4%.	High certainty: After removing the outlier, students score lower on VSAQs than MCQs, supporting a cueing effect in MCQs (recognition inflates scores).
Discrimination (Psychometric Quality)	Pooled discrimination: Fisher’s z = 1.45; *p* < 0.00001. VSAQs showed discrimination comparable to or exceeding MCQs.	High certainty: VSAQs effectively distinguish between high- and low-performing candidates.
Reliability (Internal Consistency)	Pooled reliability: Fisher’s z = 0.43. Heterogeneity: I² = 52% (moderate), indicating reasonable consistency across studies.	Moderate certainty: VSAQs maintain acceptable internal consistency comparable to MCQs; open-ended formats are not inherently unreliable.
Practice Effects (Learning Retention)	Mixed results: Earlier data Hedges’ g = 0.59 (positive effect); recent data Hedges’ g = -0.26 (non-significant trend).	Low certainty: Evidence is inconclusive on whether VSAQs improve retrieval-based learning more than MCQs; further research is needed.

## Conclusion

Very Short Answer Questions (VSAQs) provide a robust alternative to Multiple Choice Questions (MCQs) in health professions education by minimizing cueing effects and requiring more cognitively active response generation. Although students tend to score lower on VSAQs, these assessments demonstrate high discrimination and acceptable reliability, reflecting a more authentic evaluation of independent knowledge and clinical reasoning. Integrating VSAQs alongside or in place of MCQs may enhance the validity of written examinations, supporting the development of competent and self-directed learners. Further research is warranted to explore optimal implementation strategies, the impact on long-term retention, and the role of VSAQs in different educational contexts.

## Data Availability

All data generated or analyzed during this study are included in this published article and its supplementary information files.

## Abbreviations

MCQs: Multiple Choice Questions
VSAQs: Very Short Answer Questions
SMD: Standardized Mean Difference
PRISMA: Preferred Reporting Items for Systematic Reviews and Meta-Analyses
